# Investigating Flavor Enhancement Methods in NaCl-Reduced Chinese Bacon (*Larou*) by Focusing on Physicochemical Characteristics, Bacterial Diversity, and Volatiles

**DOI:** 10.3390/foods13233820

**Published:** 2024-11-27

**Authors:** Li Yang, Hongjun Li, Han Wu, Xuelian Sun, Shuyun Liu, Zhifei He

**Affiliations:** 1 College of Food Science, Southwest University, No. 2 Tiansheng Road, Beibei District, Chongqing 400715, China; yangli212223@email.swu.edu.cn (L.Y.); lihongjun@swu.edu.cn (H.L.); 15137178310@163.com (H.W.); sunxuelian1996@email.swu.edu.cn (X.S.); swulsy2020@email.swu.edu.cn (S.L.); 2 Chongqing Engineering Research Center of Regional Food, Southwest University, No. 2 Tiansheng Road, Beibei District, Chongqing 400715, China; 3 Chongqing Key Laboratory of Speciality Food Co-Built by Sichuan and Chongqing, Southwest University, No. 2 Tiansheng Road, Beibei District, Chongqing 400715, China

**Keywords:** *Larou*, NaCl, bacterial diversity, GC × GC-MS, flavor compensation

## Abstract

The higher NaCl concentration of Chinese bacon, which features a unique flavor, is a major restriction to consumption. Investigating the role of NaCl in Chinese bacon (*Larou*) would be beneficial to optimize the dosage and enhance flavor. This study was conducted to categorize *Larou* by comparing the quality of *Larou* cured with different concentrations of NaCl and then to investigate the methods of flavor enhancement of NaCl-reduced *Larou*. The results showed that, based on the differences in quality, *Larou* were categorized into three types, including the low-NaCl type (<4%, LT), the medium-NaCl type (4–8%, MT), and the high-NaCl type (>8%, HT). The vital physicochemical characteristics (PCs), predominant bacteria, and key volatile compounds (VOCs) were different for each type of *Larou*. The PCs contributing to the regulation of VOCs were total volatile basic nitrogen (TVB-N) and pH in LT, thiobarbituric acid reactive substance assay (TBARS) in MT, NaNO_2,_ and moisture content in HT. *Lactococcus* or *Lactobacillus*, *Staphylococcus*, and *Kocuria* were flavor-producing bacteria in LT, MT, and HT, respectively. Vital PCs and predominant bacteria were associated with several key aldehydes, alcohols, and esters in *Larou*. Increasing the TVB-N, TBARS, and moisture content, decreasing the pH and NaNO_2_ properly, and inoculating with *Staphylococcus* and *Kocuria* were effective methods to enhance the flavor of LT. Vital PCs and predominant bacteria are prioritized to meet most of the quality and the biosafety, although key VOCs may be sacrificed at this point.

## 1. Introduction

China is the largest producer of pork, has the largest pig population, and is the largest importer of pork from other countries [1]. According to China’s National Bureau of Statistics, pork prices have long been lower than cattle, sheep, poultry, and fishery products, laying a solid foundation for pork further processing. *Larou* (Chinese bacon) is a non-ready-to-eat fermented product with a long history in China [2]. It has become a popular pork product in the past few years due to its unique flavor [3]. These traditional fermented meat products are usually made from the sides, bellies, or backs of fresh or frozen hog flesh, which are cured and then smoked or air-dried [4]. As an essential additive in the curing process, NaCl improves the texture, safety, and flavor of foods. A high level of NaCl also promotes flavor formation, improves color, and extends shelf life [5]. Regarding health, NaCl is also an important substance in maintaining the body’s metabolism, responsible for regulating the transmission activities of blood, nerves, and muscles, regulating the water content of the body, and promoting digestion and absorption [6]. However, high sodium salt intake may induce diseases such as cardiovascular disease, primary liver cancer, and atrial fibrillation [5].

The salt content of traditional Chinese fermented meat products usually ranges from 30–100 g/kg [7]. *Larou*, a fermented meat product similar to ham and sour meat, has a similar salt content. Currently, the NaCl content of low-sodium *Larou* is in the range of 40 g/kg [8]. Many studies have been conducted to explore sodium reduction strategies for fermented meat products [7]. The most direct and effective way to reduce the addition of sodium is to reduce its concentration. However, there are problems with food quality, textural characteristics, and organoleptics [5]. Previous studies using KCl or/and calcium ascorbate as alternative sodium salts for *Larou* found that lactic acid bacteria (LAB) gradually dominated the manufacturing process and contributed to accelerated protein hydrolysis and lipid oxidation during ripening [9], but partially substituted sodium salts for *Larou* may require longer storage time to achieve better flavor [3]. Reduced-sodium production of fermented foods may also face the threat of spoilage and pathogenic microorganisms, accumulation of hazardous chemicals such as biogenic amines, nitrosamines, ethyl carbamate, and mycotoxins, flavor deficiencies, and textural deterioration [10]. The change in microbial diversity was primarily induced by reduced sodium salt, which directly or indirectly caused changes in the quality of fermented meat products. Therefore, understanding the effect of NaCl content on microbial diversity in *Larou* will allow the mining of specific microorganisms that are directly related to flavor. Cultures consisting of these microorganisms are used as starters for low-sodium-salt fermented foods to maintain the characteristic flavor of the product [11].

Aroma is one of the most important characteristics of fermented meat products. Many studies have identified dozens of VOCs from Chinese traditional *Larou* using GC-MS, contributing to the study of the characteristic aroma compounds of *Larou* [4,12,13]. Compared with one-dimensional gas chromatography, two-dimensional gas chromatography can detect volatile compounds in complex mixtures and is an advantageous tool for analyzing flavor compounds in fermented foods. Ruan et al. identified 146 VOCs in a partially sodium-salt-substituted *Larou* using GC × GC-MS [3], Liao et al. detected 184 VOCs from tank-fermented broad bean paste based on GC × GC-MS [14], Ferracane et al. using a solid-phase microextraction arrow and comprehensive two-dimensional GC-MS identified 167 VOCs from whiskey with a 6 higher sensitivity and a 5 higher reproducibility compared to conventional means of detection [15]. These works laid the methodological foundation for the application of GC × GC-MS to the identification of VOCs from *Larou*.

In this study, a *Larou* model was constructed with different NaCl concentrations, including the entire range of very low NaCl concentrations to very high NaCl concentrations, to produce *Larou* of different qualities. Color, pH, moisture content, and NaNO_2_ were used to evaluate the essential quality, and TBARS and TVB-N were used to evaluate lipid oxidation and proteolysis. High-throughput sequencing and GC × GC-MS were used to determine bacterial diversity and VOC composition. Particularly, the quality relationship among PCs, bacterial diversity, and VOCs was explored by RDA/CCA and Spearman correlation. This study aimed to elucidate two questions: (1) how to categorize the NaCl concentration of cured *Larou* based on its quality, and (2) what are the possible ways to compensate for the flavor of NaCl-reduced *Larou*.

## 2. Materials and Methods

### 2.1. Preparation of Larou with Different-Salinity

The belly meat of pork was prepared according to the previous method [16]. A total of 30 kg of fresh belly meat (purchased from Chongqing Qijin Food Corporation, Chongqing, China) from 10 Rongchang pigs (age 6 months, weighing 100–120 kg, male) was cut into long strips with a size of about 15 × 6 × 4 cm and a weight of about 0.4 kg. Twenty of these meat strips were randomly selected for curing in reference to Ruan et al. [3], with minor modifications. Specifically, the 20 pork strips were divided into five groups, each of which was supplemented with a mass ratio of 3% sucrose, 2% cooking wine, 0.15% compound phosphate II (Jiahe Food Industry Co., Suzhou, China), 0.05% sodium isoascorbate, and 0.01% sodium nitrite, but with different mass ratios of NaCl. The group without added NaCl was defined as T0, 4% NaCl as T4, 8% NaCl as T8, 12% NaCl as T12, and 20% NaCl as T20. After being thoroughly mixed, they were placed in an artificial atmospheric phenomena simulator (SRN-450, Ningbo, China) for 3 days of curing (10 °C, 70% relative humidity) and turned over every 12 h. After curing, the cured pork was soaked in 5% ordinary hawthorn firewood aroma incense liquid for 3 h. Subsequently, the pork was transferred to a climate environment at 10 °C and 70% humidity for low-temperature drying and fermentation until the 18th day. These samples were vacuum packed with PET/PE composite vacuum-packing bags (0.26 mm, 30 × 40 cm^2^) using a multifunctional vacuum packer (HZ500/2SE, Shandong Dahe Machinery Technology Co., Ltd., Weifang, China) and stored in an ultra-low temperature refrigerator (−80 °C, DW-86 W100, Qingdao, China) for consistent conditions for the determination of indicators. The experiment was completed within 1 month.

### 2.2. Sensory Evaluation

Sensory evaluation was performed according to the method of Yang et al. with some modifications [17]. Ten professionally trained panelists (6 women and 4 men; ages ranging from 20 to 30 years) evaluated the quality of *Larou* with different salinities. The bacon was first cooked in boiling water for 20 min, then cut into 2 mm slices and randomized with three-digit coding. The evaluation criteria were referenced to previous quantitative descriptive statistical tables with a twenty-point scale, and texture, color, odor, state of fat, and taste were evaluated [4]. All attributes were scored based on their corresponding descriptive terms. Water and biscuits were used to clear the palates if necessary for the panelists. The experiment was kept within 20 min.

### 2.3. Determination of PCs

The NaCl concentration was determined according to the international standard ISO-1841-1:1996 (E) [18]. Briefly, 10.000 g of minced *Larou* was mixed with 100 mL of distilled and halogen-free water, boiled in a water bath for 15 min, cooled to room temperature, and then added 2 mL of 106 g/L of [K_4_Fe (CN)_6_]·3H_2_O and 2 mL of 220 g/L of zinc acetate dihydrate, mixed thoroughly, and stood for 30 min. Subsequently, the mixture was quantitatively transferred to a 200-mL volumetric flask and filtered to obtain the supernatant. Added 5 mL of 4 mol/L HNO_3_ and 1 mL of saturated ammonium iron (III) sulfate to 20 mL of filtrate, mixed thoroughly, then added 20 mL of 0.1 mol/L AgNO_3_ and 3 mL of nitrobenzene, shaking to coagulate the precipitate. Titrated with 0.1 mol/L KSCN until a pink color appearance. The amount of Cl^−^ in *Larou* was calculated from the volume of KSCN.

The color of *Larou* was determined by removing visible fat, then stirring and mixing the muscle and measuring a* (redness), b* (yellowness), and L* (lightness) with a chroma meter WR-18 (Optoelectronics Technology Co., Ltd., Shenzhen, China).

The pH value was determined according to the Chinese standard GB5009.237-2016 [19]. 2.000 g *Larou* was mixed with a 10-fold volume of 0.1 mol/L KCl and homogenized for 1 min at 4000 rpm in an ice bath, then analyzed with a digital pH meter (Chengdu Shijifangzhou Technology Co., Ltd., Chengdu, China).

The moisture content was determined according to the international standard ISO-1442-1997 [20]. Briefly, 5.000 g to 8.000 g of minced *Larou* transferred to a dish with a 3-fold mass of dying sand. Heat the dish for 2 h in an electric gravity convection oven (DGG-9240A, Shanghai Senxin Experimental Instrument Co., Ltd., Shanghai, China) set at 103 °C. The mass before and after heating was weighed until the result of two successive weighings did not exceed 0.1%. The moisture content was calculated based on mass reduction.

The NaNO_2_ content was determined according to the Chinese standard GB5009.33-2016 [21]. Briefly, 5.000 g of minced *Larou* was mixed with 12.5 mL of 50 g/L saturated borax and 150 mL of distilled water, boiled in a water bath for 15 min, and cooled to room temperature. Then 5 mL of 106 g/L [K_4_Fe (CN)_6_]·3H_2_O was added to the mixture, shaken thoroughly, and 5 mL of 220 g/L zinc acetate was added later. The volume was quantitatively adjusted to 200 mL and left to stand for 30 min. The upper layer of fat was removed, and the supernatant was collected by filtration. 40 mL of supernatant was mixed with 2 mL of 4 g/L p-aminobenzenesulfonic acid, stood for 5 min, and then 2 g/L N-1-Naphthylethylenediamine dihydrochloride was added. The volume was quantified to 50 mL and stood for 15 min. The absorbance value was measured at 538 nm with UV-1780 (Shimadzu, Japan). The NaNO_2_ content of *Larou* was calculated from the standard curve constructed from the NaNO_2_ standard.

The TBARS was determined according to a previous method [22]. 2 g of minced *Larou* was mixed thoroughly with 3 mL 1% thiobarbituric acid and 17 mL 2.5% trichloroacetic acid-acetic acid. Subsequently, drops of butylated hydroxytoluene were added to the mixture, boiled in a water bath for 30 min, and then cooled to room temperature. The mixture was mixed with an equal amount of trichloromethane, and the supernatant was obtained by filtering. The absorbance value was measured at 532 nm with UV-1780. TBARS values were calculated by the molar extinction coefficient “9.48, 152,000 M^−1^·cm^−1^”.

The TVB-N was determined according to GB5009.228-2016 [23]. An amount of 5 g of minced *Larou* was mixed with 50 mL of distilled water and homogenized at 4000 rpm for 1 min. The supernatant was then obtained by centrifugation at 10,000 rpm for 20 min. The supernatant was distilled using an automatic Kjeldahl nitrogen analyzer (KDN-1, LeiCi, China) after addition of 1 g of MgO. An amount of 20 mL of 2% (*w*/*v*) boric acid was used to absorb the fraction and subsequently titrated to a blue-purple color with 0.1 mol/L sulfuric acid. The TVB-N content was calculated from the volume consumed.

### 2.4. Diversity Analysis of Bacteria

DNA extraction was performed according to Song et al., and bacterial diversity was characterized by amplicon sequencing [24]. The amplification primers were 515FmodF (5′-GTGYCAGCMGCCGCGGTAA-3′) and 806RmodR (5′-GGACTACNVGGGTWTCTAAT-3′) to amplify the V4 region of the microorganisms. The amplification cycle was as follows: 95 °C for 3 min, 29 cycles (95 °C for 30 s, 53 °C for 30 s, and 72 °C for 45 s), and then extension at 72 °C for 10 min. The PCR products were detected by 2% agarose gel electrophoresis, then the gel was cut and recovered by the AxyPrepDNA Gel Recovery Kit (AXYGEN, Union City, CA, USA) and eluted with Tris-HCl. Following detection and quantification with the QuantiFluor™-ST Blue Fluorescence Quantification System (Promega Biotech Co., Ltd., Madison, WI, USA), the PCR products were mixed. Illumina libraries were constructed using the TruSeq^TM^ DNA Sample Prep Kit kit (Illumina, Inc., San Diego, CA, USA). Subsequently, 2 × 300 bp paired-end sequencing was performed on the llluminaNovaSeq2000 sequencer (lllumina, Inc., USA). The PE reads obtained from sequencing were subjected to sample splitting, and then optimized data were obtained through quality control, filtering, and splicing. The optimized data were then processed using DADA2/Deblur to obtain ASV representative sequence and abundance information.

### 2.5. Determination of VOCs

A combination of headspace solid-phase microextraction (HS-SPME) and GC × GC-MS was used to analyze VOCs. The analysis was performed by referring to the method of Ruan et al. with minor modifications [3]. 2.000 g of stirred *Larou* was taken into a 15 mL headspace glass vial, and 2 μL of 0.816 g/L 2-methyl-3-heptanone was added as an internal standard. The headspace glass vials were placed in a water bath at 60 °C for 30 min and then inserted into activated SPME (75 μm CAR/PDMS, Supelco, Inc., Bellefonte, PA, USA) for 30 min to extract the VOCs. After desorption at 250 °C for 5 min on a GC × GC-MS (GCMS-QP2020NX, Shimadzu, Japan) inlet, the VOCs were separated in a GC equipped with a 1D column (DB-Wax capillary column, 30 m × 250 μm, 0.25 μm) and a 2D column (DB-17ms capillary column, 1.2 m × 180 μm, 0.18 μm). The initial column temperature was held at 40 °C for 2 min and then increased to 240 °C at a rate of 6 °C/min. The carrier gas was helium at a flow rate of 1.0 mL/min. The mass spectrometry detector uses 70 eV ionization energy. The interface temperature was 250 °C, and the ion source temperature was 230 °C. The scanning range was 40~330 *m*/*z*. Cancar Panel was used to eliminate noise from raw data, merge duplicate peaks, and identify the peaks with a signal-to-noise ratio greater than 50. The compounds with positive and negative matches less than 600 were also eliminated by similarity comparison with NIST 20 (NIST, Gaithersburg, MA, USA) and confirmed by RI. The peak areas of internal standards and VOCs were analyzed semi-quantitatively using the mass concentration of the internal standards.

### 2.6. Statistical Analysis

The means of all data were obtained by four replications and expressed as means ± SE. Statistical significance (*p* < 0.05) was determined using one-way ANOVA and Tukey’s test in IBM SPSS Statistics (SPSS Inc., Chicago, IL, USA). PCoA, HCA, Kruskal–Wallis H test bar plot, RDA/CCA analysis, and Spearman’s correlation coefficients were performed through the free online platform of Majorbio Cloud Platform (www.majorbio.com). PLS-DA, calculation of VIP, and permutation test (N = 200) were performed by SIMCA 14.1 (Umetrics, Sweden). Visualization of the correlation heatmap was achieved by OriginPro 2023 software (Origin Lab Corp., Northampton, MA, USA).

## 3. Results and Discussion

### 3.1. Sensory Evaluation

**Figure 1:**
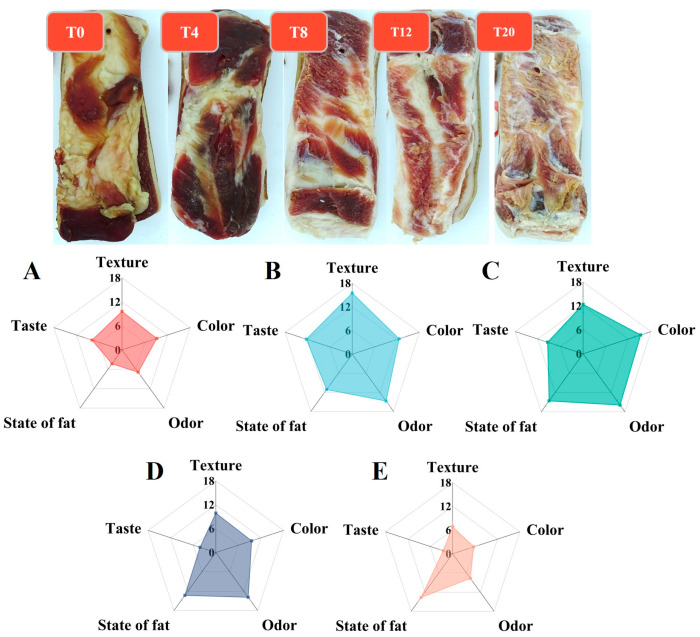
The sensory evaluation of *Larou* with different-salinity. (**A**) T0, (**B**) T4, (**C**) T8, (**D**) T12, (**E**) T20.

The sensory evaluation of *Larou* included evaluation of texture, color, odor, state of fat, and taste (Figure 1). Results showed that T4 (Figure 1B) and T8 (Figure 1C) scored higher on color and odor. And the highest texture and taste scores were obtained for T4. In addition, all the fat of *Larou* (Figure 1B–E) was transparent except T0 (Figure 1A), and the higher the NaCl concentration, the more uniform the transparency. *Larou* with higher transparency has a higher commercial value, and such transparency is related to the state of the water and the drying procedure [25]. When *Larou* was heated, the ß crystals of the fat increased and aggregated and replaced the water in the fat, resulting in a consistent refractive index and increased transparency [26]. In general, NaCl favored the formation of *Larou* flavor, but excessive NaCl affected texture, color, odor, and taste negatively. It is due to the fact that NaCl content affects the solubility of flavor compounds under the influence of the salting-in and salting-out effects. Therefore, not only can more flavor compounds be solubilized at the appropriate NaCl concentration, but also protein conformation, fat oxidation, microbial distribution, and carbohydrate catabolism can be altered to affect the overall flavor perception [7].

### 3.2. Analysis of PCs

As shown in Table 1, *Larou* exhibited an actual NaCl concentration that was not consistent with the amount of NaCl added. This may mean the NaCl on the surface of *Larou* was lost due to liquid smoking, and only the NaCl that penetrated into the interior of the meat was retained. It is helpful to analyze the PCs, microbial flora, and flavor by understanding the actual NaCl concentration of *Larou*.

The color, especially a*, of fermented meat products is a factor that influences consumer purchase because it can indicate the appearance of the product [27]. a* is associated with microbial production of NO, which interacts with the heme center and binds the myoglobin ferrous heme-iron to form nitrosomyoglobin, the red pigment of fermented meat [28]. As shown in Table 1, a* exhibited the maximum value of 9.73 in T4. Gu et al. concluded that sausage, another traditional fermented meat from China, with higher L* and a* showed more attractiveness [29]. For spontaneously fermented *Larou*, however, achieving a higher L* and a* simultaneously is impossible, potentially due to textural and microbiological differences resulting from varying NaCl concentrations. b* is the color associated with fat. NaCl affects lipid degradation by influencing lipase activity, which in turn affects the color of fat [30]. Overall, the color of spontaneously fermented bacon is optimal when the actual NaCl concentration is between 3 and 7%.

During fermentation, the pH value decreased gradually with the increase in NaCl concentration until it was maintained at about 6.0 after the NaCl concentration exceeded 7.05% (the actual NaCl concentration of T8). Whereas, the moisture content showed an opposite trend with pH, which increased to about 30% after the NaCl concentration exceeded 7.05% (Table 1). Song et al. also observed this phenomenon, where the pH of *Larou* was around 6.0 after the NaCl concentration exceeded 8%, while the moisture content was maintained at around 31% during the earlier fermentation [24]. Decreased pH of fermented meat products may be caused by degradation of fat and fermentation of carbohydrates by endogenous enzymes and microbial-produced enzymes, where some metabolites such as short-chain fatty acids, acetic acid, butyric acid, propionic acid, and lactic acid could acidify the matrix [31]. Notably, higher NaCl concentration was accompanied by higher water content, which may be beneficial for flavor development in fermented meat products. In the hypersaline condition, enzymes in some halophilic archaea show activity by adjusting their structure to possess acidic amino acid residues, and the negatively charged amino acids on the surface of the enzyme need to bind to hydrated cations to adapt to the hypersaline environment, thus enhancing proteolysis of the substrate [32].

NaNO_2_ showed a maximum value of 1.40 μg/g in T12 (Table 1). NaNO_2_ is commonly used as a color protectant, antioxidant, preservative, and flavor booster in meat fermentation. The excessive quantity of NaNO_2_ will be degraded by acids and enzymes, as well as non-acidic and non-enzymatic compounds in bacteria [33]. Since all the samples in this experiment were added with equal amounts of NaNO_2_, the different concentrations of NaNO_2_ in the fermented *Larou* may be due to the presence of microorganisms that degrade NaNO_2_, but the microorganisms in the high-NaCl *Larou* were not as competent as those in the low-NaCl *Larou*.

TBARS is a measure of lipid oxidation in meat products, where moderate lipid oxidation facilitates aroma development, while excessive lipid oxidation generates off-flavors [34]. The differences in TBARS were not significant in all groups, except for the T4 (*p* < 0.05). It has been demonstrated that pro-oxidation of lipids in meat occurred at lower NaCl concentrations. Triglyceride hydrolase, protein kinase, and alkaline phosphatase activities were highest at 3% NaCl concentration when lipid oxidation was elevated [35].

TVB-N is a reflection of the content of organic amines and ammonia induced by the decomposition of proteins and other nitrogenous constituents due to microbial activity, which is one of the key attributes in characterizing the freshness of meat [36]. The TVB-N in *Larou* gradually decreased with increasing NaCl concentration, with the T0 group showing a high TVB-N value of 28.6 mg/100 g. TVB-N decreased significantly after the NaCl concentration exceeded 8%, a result consistent with Song et al. [24]. This is presumably explained by the fact that *Larou* with a higher NaCl concentration has a lower pH, and the low pH suppressed the transition of glycogen-dependent microorganisms to protein-degrading microorganisms [37]. The phenomenon is also deepened by high NaCl concentrations, which inhibit the growth of undesirable microorganisms.

### 3.3. Analysis of Bacterial Diversity

To investigate the effect of NaCl on bacterial composition, we analyzed the bacterial diversity by high-throughput sequencing. A coverage of 1.00 for each group was obtained, indicating that the sequencing results yielded adequate data to reflect the composition of microorganisms in *Larou* (Table 2). The ACE was used to reflect community richness, Shannon and Simpon were used to reflect diversity, and Shannoneven was used to reflect evenness [7]. No significant differences in ACE were found in all *Larou*, indicating that all groups were rich in microorganisms, whereas the bacterial diversity was significantly higher (*p* < 0.05) in T0, T4, and T8 than in T12 and T20. Distinctively, the community evenness of T4 was lower than that of T0, T8, T12, and T20, indicating that NaCl was able to influence microbial composition, especially at low NaCl concentrations.

**Figure 2:**
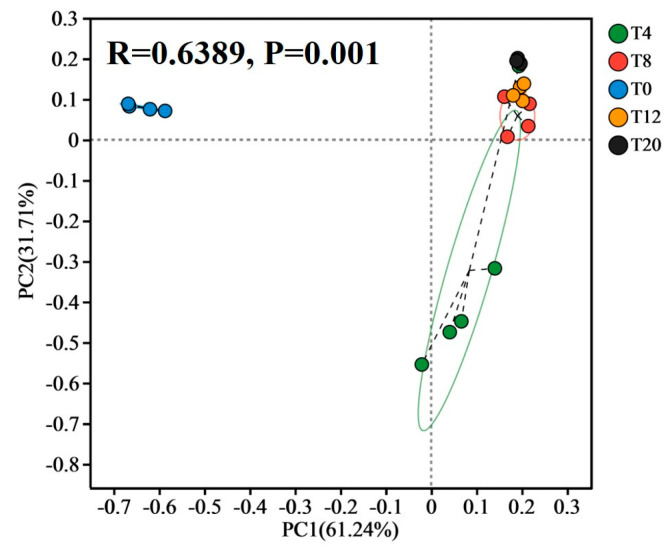
Principal co-ordinates analysis (PCoA) analysis of bacterial composition. 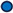, T0; 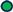, T4; 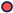, T8; 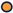, T12; 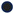, T20. The circles represent the 95% confidence intervals between samples, and the dashed lines are the connections between the samples and the center of the confidence intervals.

**Figure 3:**
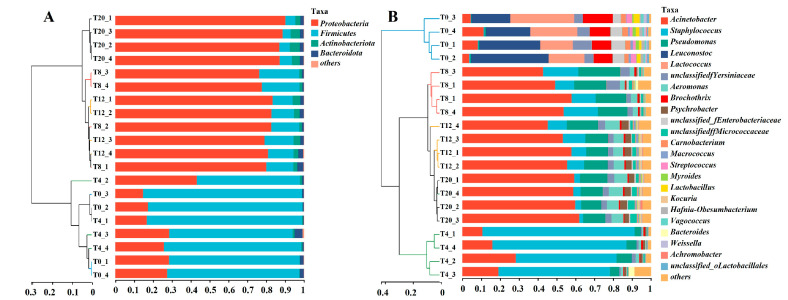
Changes in the structure of bacteria at *Larou* with different NaCl concentrations. Bacterial community composition at the (**A**) phylum and (**B**) genus level.

The bacterial composition of *Larou* with NaCl was significantly different from that without NaCl, as analyzed by PCoA (Figure 2). In addition, the NaCl also affected the bacterial composition of *Larou*, as evidenced by the fact that the bacterial composition of T4 was significantly different from that of T8, T12, and T20. T8 exhibited the highest similarity in bacterial composition to that of T12. The ASV from different *Larou* was taxonomized at the phylum and genus level. Taxonomical assignment at the phylum level revealed a total of 4 phyla. *Protobacteria*, *Firmicutes*, *Actinobacteriota*, and *Bacteroidota* were the major bacterial phyla in terms of relative abundance. All groups were detected for all four phyla, accounting for more than 99% of the overall abundance, except for T0, where *Actinobacteriota* was not detected (Figure 3A). Relative abundance of Firmicutes and *Proteobacteria* was significantly affected by NaCl concentration, with higher NaCl concentrations resulting in lower Firmicutes. Taxonomical assignment at the genus level revealed 23 genera with abundances greater than 0.01. Major differences were also observed in the genus-level composition of bacteria between the groups where NaCl was added (T4, T8, T12, and T20) and without NaCl (T0) (Figure 3B).

### 3.4. Changes in Bacterial Abundance

**Figure 4:**
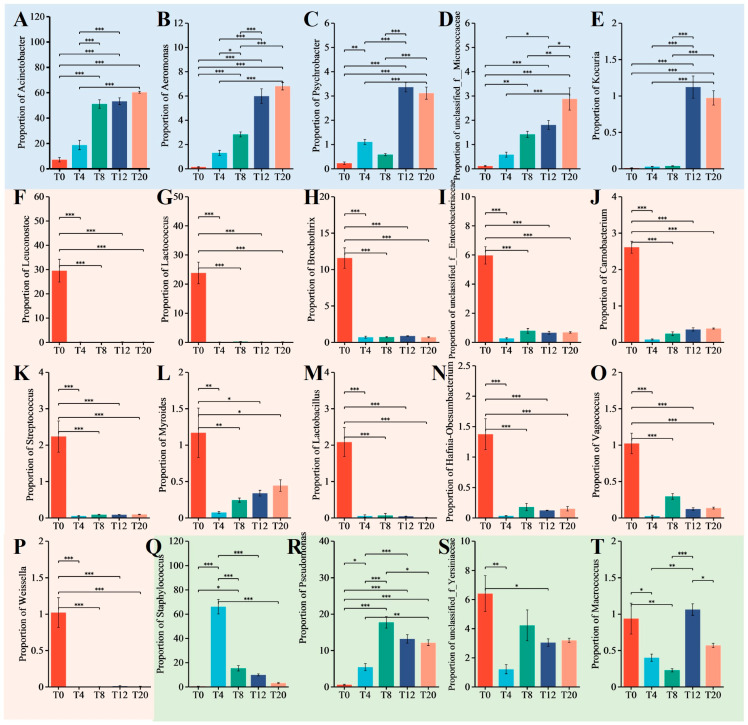
Kruskal–Wallis H test bar plot of bacterial community. Multiple validation corrections were made using false discovery rate (FDR) and post-hoc tests were made using Tukey–kramer (0.95). (**A**) *Acinetobacter*; (**B**) *Aeromonas*; (**C**) *Psychrobacter*; (**D**) *Unclassified_f_Micrococcaceae*; (**E**) *Kocuria*; (**F**) *Leuconostoc*; (**G**) *Lactococcus*; (**H**) *Brochothrix*; (**I**) *Unclassified_f_Enterobacteriaceae*; (**J**) *Carnobacterium*; (**K**) *Streptococcus*; (**L**) *Myroides*; (**M**) *Lactobacillus*; (**N**) *Hafnia-Obesumbacterium*; (**O**) V*agococcus*; (**P**) *Weissella*; (**Q**) *Staphylococcus*; (**R**) *Pseudomonas*; (**S**) *Unclassified_f_Yersiniaceae*; (**T**) *Macrococcus*. * *p* < 0.05; ** *p* < 0.01; *** *p* < 0.001.

Changes in bacterial abundance reflect the shaping effect of NaCl on the microbiota. Abundance of *Acinetobacter* (Figure 4A), *Aeromonas* (Figure 4B), *Psychrobacter* (Figure 4C), *unclassified_f_Micrococcaceae* (Figure 4D), and *Kocuria* (Figure 4E) increased significantly (*p* < 0.05) with increasing NaCl concentration. They were able to survive at high NaCl concentrations. *Acinetobacter* and *Psychrobacter* are core microorganisms in *Larou* and raw fermented sausages and may be associated with flavor production [2,38]. *Acinetobacter* readily utilizes organic acids and amino acids as the sole source of carbon, nitrogen, and energy, and, as a lipolytic bacteria, it might contribute to the VOCs of meat; however, it is generally believed that some of these VOCs are the source of off-flavors in fermented meat products [39,40]. *Aeromonas* can ferment glucose, fructose, maltose, and trehalose into acids and/or gas as a source of odors for fermented foods, but *Aeromonas* is an opportunistic pathogen and has also been considered a potential foodborne pathogen that is detrimental to food production [41]. *Kocuria* is used in food fermentation for its prominent catalase and protease activities to increase volatiles in foods. The safety of *Kocuria* is also often feared due to issues of resistance to antimicrobials [42].

Abundance of *Leuconostoc* (Figure 4F), *Lactococcus* (Figure 4G), *Brochothrix* (Figure 4H), *unclassified_f_Enterobacteriaceae* (Figure 4I), *Carnobacterium* (Figure 4J), *Streptococcus* (Figure 4K), *Myroides* (Figure 4L), *Lactobacillus* (Figure 4M), *Hafnia-Obesumbacterium* (Figure 4N), *Vagococcus* (Figure 4O), and *Weissella* (Figure 4P) decreased rapidly and significantly (*p* < 0.05) with the increasing of NaCl concentration. They preferred to survive at low NaCl concentrations. Interestingly, although the LAB had the highest abundance in the T0, the pH was higher (7.07), which could be attributed to the lower absolute abundance of LAB with less effect on the acidity of *Larou*. For fermented meat products, *Leuconostoc* is considered a spoilage bacterium due to the slime forming, *Lactococcus* is a homofermentative LAB that could grow below 7 °C, *Streptococcus* does not form endospores and has an exopolysaccharide production capacity with an optimal growth temperature range of 35–42 °C, and *Weissella* is a heterofermentative bacteria [43]. *Lactobacillus* is a microaerophilic to strictly anaerobic LAB with rapid acid production and a wide range of probiotic properties that can also be used for meat fermentation [44]. *Vagococcus* is a widely distributed motile LAB; the role of *Vagococcus* in food spoilage and the production of metabolic compounds is still unclear [45]. *Enterobacteriaceae* (*unclassified_f_Enterobacteriaceae* and *Hafnia-Obesumbacterium*) is responsible for meat spoilage [46]. *Myroides* are ubiquitous probable pathogens in the environment with less well-documented use in food. *Brochothrix* is a common spoilage microorganism of meat and meat products stored at chilled temperatures and also leads to the formation of slime textures on cured and processed meat products. *Carnobacterium* is the predominant microorganism in chilled vacuum- or modified atmosphere-packed meat and seafood, as well as dairy products, and is associated with the production of sensory-spoiled products [43].

The abundance of *Staphylococcus* (Figure 4Q) and *Pseudomonas* (Figure 4R) was minimal in T0 and increased significantly (*p* < 0.05) after the addition of NaCl, with the highest abundance of *Staphylococcus* in T4 and *Pseudomonas* in T8. They had a suitable concentration of NaCl for survival. *Staphylococcus* is a microhalophilic bacteria in which coagulase-positive phenotypes such as *Staphylococcus aureus* are usually considered pathogenic bacteria, while coagulase-negative phenotypes such as *S. xylosus*, *S. saprophyticus*, and *S. carnosus* are often used in meat fermentation due to their prominent catalase and protease activities [47]. *Pseudomonas* is a common bacterium in fermented meat that can use hydrocarbons as a carbon source to produce rhamnolipids for food additives, whereas it might cause nosocomial infections [2,48]. The abundance of *unclassified_f_Yersiniaceae* (Figure 4S) and *Macrococcus* (Figure 4T) was the lowest in T4 and T8, respectively, suggesting that these two bacteria can survive well in low or high NaCl environments. *Yersiniace* is a pathogenic bacteria, and *Macrococcus* is usually unrecognized as a human pathogen. For example, *Macrococcus caseolyticus* can hydrolyze casein for use in dairy and meat fermentation [49].

In summary, LAB was the predominant bacterium in *Larou* at low NaCl concentrations (<4%), most of which were spoilage bacteria or unfavorable for sensory formation. *Lactococcus* and *Lactobacillus* were expected to be used in fermentation due to a better adaptation to the production conditions of *Larou*, a good metabolic character, and a possible probiotic effect. At high NaCl concentrations (>12%), the growth of halophilic spoilage bacteria was found to be massive, and *Kocuria* was expected to be used in *Larou* fermentation due to its catalase and protease activities. Pathogenic bacteria were found to be more prevalent at moderate NaCl concentrations (4%–8%), whereas *Staphylococcus*, due to catalase and protease activities, are commonly used in the *Larou* fermentation.

### 3.5. Analysis of VOCs

The content of NaCl impacted the PCs and bacterial activity of *Larou* directly or indirectly, thus affecting the composition of VOCs. VOCs from *Larou* at five NaCl concentrations were analyzed by GC × GC-MS, and a total of 146 compounds were detected, including 20 phenolic compounds, 28 esters, 13 acids, 16 ketones, 28 alcohols, 7 aromatic compounds, 3 furans, 7 aldehydes, 5 hydrocarbons, and 19 other compounds (Supplementary Table S1).

**Figure 5:**
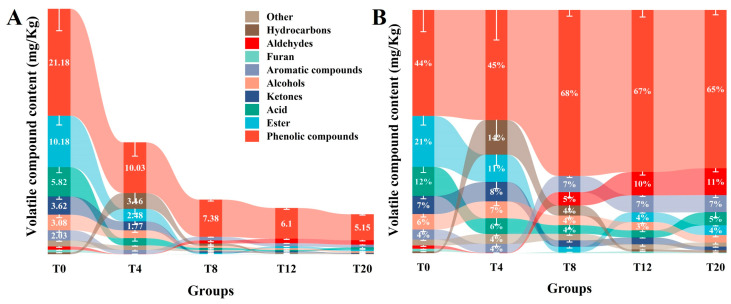
Content of VOCs in *Larou* at different NaCl concentrations. (**A**) semi-quantitative content based on internal standards; (**B**) percentage content.

In terms of content, phenolic compounds, esters, and acids were the most abundant compounds (Figure 5A). Phenolic compounds are primarily derived from smoking and impart woody/smoky, green, fruity, sweet, and vegetable aromas to meat products [50]. Interestingly, the content of phenolic compounds continued to decrease with increasing NaCl concentration, from 21.18 mg/kg at T0 to 5.18 mg/kg at T20. It might be related to the high level of NaCl that weakened the mass transfer process on the bacon surface, and the dry surface formed by *Larou* prevented the adsorption of VOCs. Esters are generated by esterification of fatty acids with alcohols and by microbial metabolism, where short-chain esters (C1–C10) impart a fruity aroma to fermented meat products and long-chain esters produce the fatty flavor [51]. The content of esters remained relatively stable after decreasing from 10.18 mg/kg at T0 to 0.24 mg/kg at T8. The disappearance of some short-chain esters, such as 2,2-dimethyl-butanoic acid, methyl ester, hexanoic acid, ethyl ester, propanoic acid, and 2-hydroxy-ethyl ester, and the appearance of a number of medium and long-chain esters, such as 13-tetradecynoic acid, methyl ester, nonanoic acid, methyl ester, and 2-octyl-cyclopanetradecynoic acid, may be a specific indication of a change in *Larou’*s flavor. Medium- and long-chain fatty acids produced by lipase-catalyzed production of triglycerides and phospholipids, or short-chain fatty acids produced by pyruvate metabolism and lipoxygenase-catalyzed, are the main sources of acids [52]. NaCl appeared to intensely affect the content of acids, which significantly decreased from 5.82 mg/kg at T0 to 1.42 mg/kg at T4 once NaCl was added and then remained relatively constant.

The aroma of fermented meat products results from an ideal and well-balanced composition of several VOCs, and it is particularly important that only those with concentrations above the olfactory threshold are odor-active [51]. In terms of percentage contents, phenolic compounds, esters, and acids were important VOCs in T0, while hydrocarbons, aldehydes, and aromatic compounds became important VOCs with increasing NaCl concentrations (Figure 5B). Hydrocarbons probably originate from the auto-oxidation of unsaturated fatty acids, which contribute slightly to the overall flavor due to a high olfactory threshold [53]. Aldehydes have a low olfactory threshold, imparting a grassy aroma to fermented meats at low concentrations and a rancid odor at high concentrations [50]. The percentage contents of the aldehydes were significantly elevated when the added NaCl concentrations exceeded 8%, reaching 11% in T20, where the nonanal was significantly increased. Nonanal is derived from the lipid oxidation of oleic acid and provides a sweet and fruity flavor that can enhance the flavor of fermented meat products [50]. Aromatic compounds may originate from the roasting or fumigation process, and a number of aromatic compounds have been shown to contribute stable and woody aromas [54,55]. Different from phenolic compounds, aromatic compounds had a stabilized content in every NaCl-added group, although their content was significantly lower than that of T0.

### 3.6. Discriminant Analysis of VOCs

**Figure 6:**
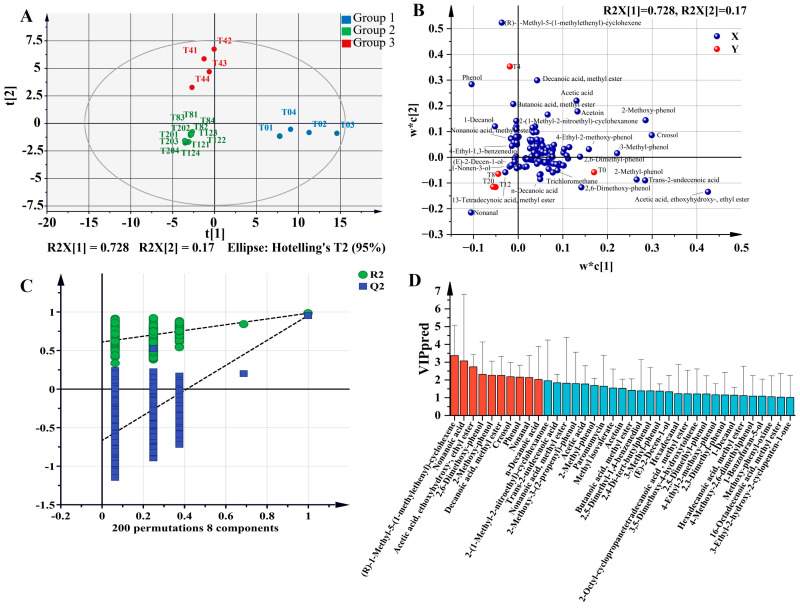
PLS-DA to analyze VOCs from *Larou* with different NaCl concentrations. (**A**) Score plot (R2X(cum) = 0.982, R2Y(cum) = 0.979, Q2(cum) = 0.877), (**B**) loading diagram, (**C**) permutation plot (N = 200) to check the validity and the degree of overfit for the PLS-DA, and (**D**) variable important in projection (VIP) values. The dashed lines in Figure C represent the connections between the original model and the Y-permuted models.

As a result of PLS-DA of VOCs, *Larou* with different NaCl concentrations were categorized into three groups: group1 including T0, group2 including T8, T12, and T20, and group3 including T4 (Figure 6A). According to the permutation test (Figure 6C), the Q2-values to the left were lower than the original points to the right, and the blue regression line of the Q2-points intersects the vertical axis below zero, indicating that the PLS-DA model was not overfitting. The loading plot by PLS-DA showed that (R)-1-methyl-5-(1-methylethenyl)-cyclohexene, phenol, butanoic acid, methyl ester, decanoic acid, methyl ester, and 1-decanol were characterized VOCs that distinguished T4 from the other groups; nonanal, 13-tetradecynoic acid, methyl ester, 1-nonen-3-ol, and (E)-2-decen-1-ol were the characteristic VOCs of T8, T12, and T20 from the other groups; and acetic acid, ethoxyhydroxy-,ethyl ester, trans-2-undecenoic acid, 2-methyl-phenol, and 2,6-dimethoxy-phenol were characterized VOCs that distinguished T0 from the other groups (Figure 6B). In order to objectively identify the VOCs responsible for the differences of *Larou*, X variables with VIP greater than 1 were screened out for their possible contribution to the model. In Figure 6D, we obtained a total of 37 VOCs with VIP greater than 1. All kinds of VOCs were included except furans. Phenolic compounds, esters, and acids were the dominant kinds that caused differences in the VOCs of *Larou*, suggesting that liquid smoke, fat oxidation, and microbial activity had a strong influence on flavor.

### 3.7. Correlation Analysis of PCs, Bacterial Diversity, and VOCs

**Figure 7:**
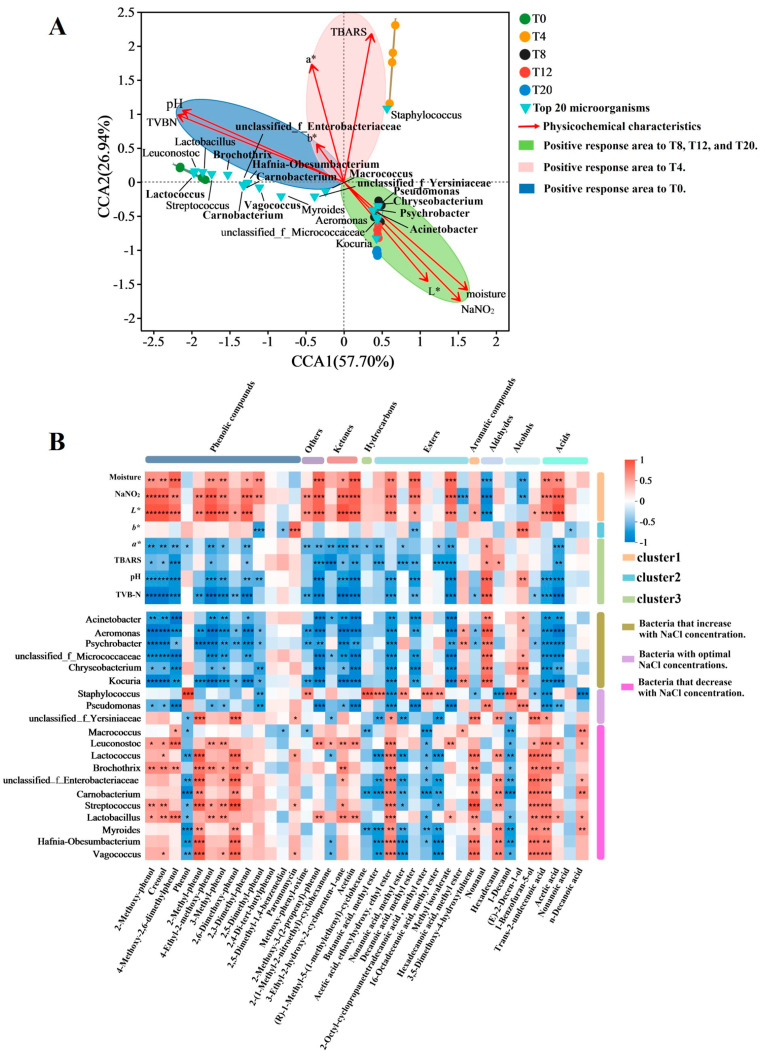
RDA/CCA analysis of PCs and bacteria (**A**) and correlation heatmap of bacteria and VOCs with VIP > 1 (**B**). In Figure 5B, R-values are indicated by different colors and *p*-values are indicated by asterisks, where * indicates 0.01 < *p* ≤ 0.05, ** 0.001 < *p* ≤ 0.01, and *** *p* ≤ 0.001.

A series of biochemical, microbiological, and chemical changes will take place during the meat fermentation process, which will affect the taste, color, and aroma [56]. Therefore, it is important to analyze the correlation of PCs, bacterial diversity, and VOCs in *Larou* to investigate the role of NaCl in flavor. In this study, the RDA/CCA was used to explore the drivers (PCs) affecting the distribution of bacteria, and the correlation coefficients between VOCs with VIP > 1 and the top 20 abundance bacteria and PCs were analyzed using the Spearman correlation test, respectively. The results are shown in Figure 7. Consistent with the results of the PCoA and PLS-DA analyses, the chi-square distances distributed the five samples in different quadrants and classified them into three categories, with T0 (category 1) being distributed in the second and third quadrants, T4 (category 2) in the fourth quadrant, and T8, T12, and T20 (category 3) in the first quadrant. In addition, TVB-N, pH, b*, and a* values were distributed in the third quadrant, TBARS in the second quadrant, and L*, NaNO_2_, and moisture content in the first quadrant. *Lactococcus*, *Leuconostoc*, *Lactobacillus*, *Streptococcus*, *Brochothrix*, *Carnobacterium*, *Hafnia-Obesumbacterium*, *unclassified_f_Enterobacteriacea*, *Vagococcus*, *Myroides*, *unclassified _f_Yersiniaceae*, and *Macrococcus* were found to be closest to category 1 (T0), which indicated that TVB-N, pH, b*, and a* affected their distribution at a low NaCl concentration (<4%), especially TVB-N and pH. In particular, *Staphylococcus* was closest to category 2 (T4), and the angles to both TBARS and a* were acute, demonstrating that, at a medium NaCl concentration (~4%), TBARS and a* were PCs that affected the distribution of *Staphylococcus*, especially TBARS. In contrast, *Kocuria*, *Aeromonas*, *unclassified_f_Micrococcaceae*, *Acinetobacter*, *Psychrobacter*, and *Pseudomonas* were closest to category 3 (T8, T12, and T20), demonstrating that L*, NaNO_2_, and moisture content were the PCs that affected the distribution of those bacteria, especially NaNO_2_ and moisture content, at a high NaCl concentration (>4%).

PCs, on the one hand, directly affected the generation of VOCs; on the other hand, they indirectly affected the generation of VOCs by affecting the distribution of microorganisms. The correlation coefficients between VOCs with VIP > 1 and the top 20 abundance bacteria and PCs are visualized in Figure 7B. In addition to phenolic compounds, NaNO_2_, moisture content, and L* were positively correlated with ketones (3-ethyl-2-hydroxy-2-cyclopenten-1-one and acetoin), esters (acetic acid, ethoxyhydroxy, ethyl ester, decanoic acid, methyl ester, and methyl isovalerate), and acids (trans-2-undecenoic acid and acetic acid), while a*, TBARS, pH, and TVB-N were positively correlated with aldehydes (nonanal) and alcohols ((E)-2-decen-1-ol), which reflected the fact that NaCl concentrations shaped the profiles of VOCs by influencing PCs.

Hexadecanoic acid, methyl ester, nonanal, and (E)-2-decen-1-ol are all important VOCs in traditional Chinese bacon [4,17]. In Figure 7B, hexadecanoic acid, methyl ester, nonanal, and (E)-2-decen-1-ol were positively correlated with bacteria that were survivable at high NaCl concentrations (>8%), with nonanal positively correlating with all five bacteria (*p* < 0.01). While hexadecanoic acid, methyl ester, and (E)-2-decen-1-ol were positively correlated with *Aeromonas* and *Kocuria* (*p* < 0.01). Consequently, *Kocuria* appeared to be a favored candidate for fermentation in terms of safety. Studies have used *Kocuria rhizophila* in the fermentation of dry-cured hams and found that it contributed to the development of color and flavor [57]. At the medium-NaCl of *Larou* (4–8%), *Staphylococcus* had a more significant correlation (*p* < 0.01) with esters (butanoic acid, methyl ester, octyl-cyclopropanetetradecanoic acid, methyl ester, and 16-octadecenoic acid, methyl ester) and alcohols (1-decanol) compared to *Pseudomonas*. Previous studies have shown that mixed fermentation of *S. cohnii WX_M8* and *S. saprophyticus MY_A10* can harmonize flavors by altering the amount or types of esters and alcohols in *Larou* [17]. However, *Staphylococcus* required manual selection of those phenotypically safe types for use in meat fermentation. At a low NaCl concentration (<4%), bacteria with higher abundance were mainly positively correlated with ketones (3-ethyl-2-hydroxy-2-cyclopenten-1-one and acetoin), aromatic compounds (3,5-dimehtoxy-4-hydroxytoluene), alcohol (1-benzofuran-5-ol), and acid (trans-2-undecenoic acid) (*p* < 0.05).

## 4. Conclusions

This study is the first to explore the changes in quality of *Larou* under different NaCl concentrations. Those *Larou* samples were categorized into three types according to the PCs, bacterial diversity, and VOCs. The curing NaCl concentration < 4% was the LT, which featured higher TVB-N and pH, as well as acetic acid, ethoxyhydroxy-, ethyl ester, trans-2-undecenoic acid, 2-methyl-phenol, and 2,6-dimethoxy-phenol VOCs and a diverse LAB. The curing NaCl concentration of 4–8% was the MT, which featured higher TBARS and a* and lower b*, as well as key VOCs (R)-1-methyl-5-(1-methylethenyl)-cyclohexene, phenol, butanoic acid, methyl ester, decanoic acid, methyl ester, and 1-decanol, and higher abundances of *Staphylococcus* and *Pseudomonas*. And curing NaCl concentration >4% was the HT, which featured higher NaNO_2_, moisture content, and L*, as well as nonanal, 13-tetradecynoic acid, methyl ester, 1-nonen-3-ol, and (E)-2-decen-1-ol VOCs and higher abundances of *Acinetobacter*, *Aeromonas*, *Psychrobacter*, and *Kocuria*.

NaCl is a necessary additive, but *Larou* with different salinity featured distinct flavor advantages. In order to meet the production requirements of low-NaCl fermented meat products, many measures can be used to improve or preserve flavor. In terms of production, artificial optimization of the production process can be considered to control PCs to modulate the flavor of low-NaCl *Larou*. For example, enrichment of VOCs and modulation of microbial diversity by increasing the TVB-N (protein degradation), TBARS (fat oxidation), and moisture content as well as decreasing the pH and NaNO_2_ of LT properly. In terms of adjuncts, starters that aid in flavor generation can be used. *Lactococcus* or *Lactobacillus*, *staphylococci*, and *Kocuria* could be used in the mixed fermentation of *Larou* to restore or enhance the flavor of *Larou* as much as possible. In terms of actual manufacturing, optimization of NaCl dosage should take into account the “give and take” rule between PCs, VOCs, and microbial safety in order to cope with the deterioration of PCs and the increase of spoilage and pathogens caused by the reduction of NaCl.

## Figures and Tables

**Table 1:** PCs of *Larou* with different NaCl concentration.

Indicators	Sample Codes					*p* Value
	T0	T4	T8	T12	T20	
NaCl (%)	0.42 ± 0.08 ^e^	3.23 ± 0.03 ^d^	7.05 ± 0.01 ^c^	7.59 ± 0.07 ^b^	8.81 ± 0.01 ^a^	<0.0001
a*	8.84 ± 0.55 ^a^	9.73 ± 0.53 ^a^	8.83 ± 0.93 ^a^	8.79 ± 0.37 ^a^	4.72 ± 0.80 ^b^	<0.0001
b*	6.97 ± 1.44 ^a^	7.58 ± 0.42 ^a^	2.93 ± 1.05 ^b^	5.28 ± 0.94 ^b^	8.47 ± 1.08 ^a^	0.01530
L*	1.05 ± 0.21 ^d^	6.23 ± 0.90 ^cd^	11.20 ± 1.06 ^bc^	14.85 ± 1.85 ^b^	35.09 ± 2.51 ^a^	<0.0001
pH	6.47 ± 0.02 ^a^	6.21 ± 0.02 ^b^	6.03 ± 0.02 ^c^	6.03 ± 0.01 ^c^	6.05 ± 0.01 ^c^	<0.0001
Moisture content (%)	17.61 ± 2.05 ^b^	21.40 ± 0.25 ^b^	32.71 ± 0.44 ^a^	31.63 ± 1.01 ^a^	30.43 ± 1.07 ^a^	<0.0001
NaNO_2_ (μg/g)	0.59 ± 0.01 ^d^	0.73 ± 0.004 ^c^	1.16 ± 0.03 ^b^	1.40 ± 0.02 ^a^	1.26 ± 0.03 ^b^	<0.0001
TBARS (mg MDA/kg minced *Larou*)	0.74 ± 0.01 ^b^	0.97 ± 0.11 ^a^	0.68 ± 0.01 ^b^	0.73 ± 0.01 ^b^	0.66 ± 0.02 ^b^	0.0078
TVB-N (mg/100 g)	28.60 ± 1.40 ^a^	14.54 ± 0.27 ^b^	9.42 ± 0.61 ^c^	7.70 ± 0.10 ^cd^	5.98 ± 0.24 ^d^	<0.0001

All data is expressed as means ± SE (*n* = 4), except a*, b*, and L* are expressed as means ± SE (*n* = 6). ^a–e^ Means with different superscripts within the rows are significantly different (*p* < 0.05).

**Table 2:** Alpha diversity index of bacterial community composition.

Indicators	Sample Codes					*p* Value
	T0	T4	T8	T12	T20	
ACE	90.25 ± 6.85 ^a^	214.00 ± 96.14 ^a^	184.00 ± 46.23 ^a^	187.30 ± 21.83 ^a^	156.30 ± 10.01 ^a^	0.4738
Shannon	2.76 ± 0.08 ^ab^	2.48 ± 0.24 ^b^	2.85 ± 0.13 ^ab^	3.23 ± 0.04 ^a^	3.12 ± 0.03 ^a^	0.0059
Simpson	0.11 ± 0.01 ^ab^	0.19 ± 0.04 ^a^	0.12 ± 0.02 ^ab^	0.07 ± 0.01 ^b^	0.08 ± 0.01 ^b^	0.0036
Shannoneven	0.61 ± 0.01 ^a^	0.48 ± 0.03 ^b^	0.56 ± 0.01 ^a^	0.62 ± 0.01 ^a^	0.62 ± 0.01 ^a^	<0.0001
Coverage	1.00 ^a^	1.00 ^a^	1.00 ^a^	1.00 ^a^	1.00 ^a^	-

Data are expressed as means ± SE (*n* = 4). ^a,b^ Means with different superscripts within the rows are significantly different (*p* < 0.05).

## Data Availability

The original contributions presented in the study are included in the article/Supplementary Materials, further inquiries can be directed to the corresponding author.

## Supplementary Materials

The following supporting information can be downloaded at: https://www.mdpi.com/article/10.3390/foods13233820/s1, Table S1. Identification and quantitative of VOCs content in *Larou* with different NaCl concentrations.
