# Supplementary material for: Investigating Flavor Enhancement Methods in NaCl-Reduced Chinese Bacon (Larou) by Focusing on Physicochemical Characteristics, Bacterial Diversity, and Volatiles
Source: Foods. 2024 Nov 27;13(23):3820. doi: 10.3390/foods13233820 (PMC11640370; doi:10.3390/foods13233820)
Supplement: Supplementary file 1 [file foods-13-03820-s001.zip › foods-3252271-supplementary.pdf]

Supplementary table S1

Identification and quantitative of VOCs content in *Larou* with different NaCl concentrations.

Volatile compounds	Relative content (mg/Kg)					*P* value
	T0	T4	T8	T12	T20	
**Phenolic compounds (20)**						
2-Methoxy-phenol	4.74 ± 0.52[a]	2.53 ± 0.48[b]	1.55 ±0.02[bc]	1.11 ±0.01[c]	1.98 ±0.02[c]	< 0.0001
Creosol	5.14 ± 0.57[a]	2.44 ± 0.37[b]	1.64 ± 0.03[b]	1.48 ±0.03[b]	1.11 ±0.01[b]	< 0.0001
2,6-Dimethyl-phenol	0.82 ± 0.08[a]	0.19 ± 0.02[b]	0.07 ± 0.01[b]	0.05 ±0.01[b]	0.08 ±0.01[b]	< 0.0001
4-Methoxy-2,6-dimethylphenol	0.22 ± 0.01[a]	0.10 ± 0.01[b]	-	0.05 ±0.01[c]	-	< 0.0001
Phenol	0.05 ± 0.01[c]	1.47 ± 0.13[a]	0.81 ± 0.02[b]	0.65 ±0.01[b]	0.55 ±0.01[b]	< 0.0001
2-Methyl-phenol	2.74 ± 0.26[a]	-	0.05 ± 0.01[b]	-	-	< 0.0001
2,3,5-Trimethyl-phenol	0.08 ± 0.01[a]	0.05 ± 0.01[ab]	0.03 ± 0.01[b]	0.03 ±0.01[b]	-	< 0.0001
4-Ethyl-2-methoxy-phenol	1.39 ± 0.12[a]	0.62 ± 0.08[b]	0.44 ± 0.01[bc]	0.39 ±0.01[bc]	0.28 ±0.02[c]	< 0.0001
3-Methyl-phenol	2.75 ± 0.23[a]	1.13 ± 0.18[b]	0.84 ± 0.01[b]	0.79±0.04[b]	0.70 ±0.02[b]	< 0.0001
2,6-Dimethoxy-phenol	1.99 ± 0.17[a]	0.78 ± 0.26[b]	1.24 ± 0.01[b]	1.04 ±0.03[b]	0.89 ±0.03[b]	< 0.0001
2-Methoxy-4-propyl-phenol	0.17 ± 0.01[a]	0.08 ± 0.01[b]	0.06 ± 0.01[b]	0.04 ± 0.01[b]	0.04 ± 0.00[b]	< 0.0001
2,3-Dimethyl-phenol	0.40 ± 0.09[a]	0.19 ± 0.02[b]	0.16 ± 0.01[b]	0.10 ±0.01[b]	0.05 ±0.01[b]	0.001
2-Methoxy-3-(2-propenyl)-phenol	0.08 ± 0.00[a]	0.04 ± 0.01[b]	0.01 ± 0.01[c]	-	0.07 ±0.01[a]	< 0.0001
4-Methoxy-3-(methoxymethyl)-phenol	0.09 ± 0.01[a]	0.02 ± 0.01[b]	0.01 ± 0.01[b]	-	-	< 0.0001
2,5-Dimethyl-phenol	0.15 ± 0.02[a]	0.15 ± 0.05[a]	0.19 ± 0.01[a]	0.13 ±0.01[a]	0.16 ±0.03[a]	0.737
2,4-Di-tert-butylphenol	0.01 ± 0.01[b]	0.02 ± 0.00[b]	0.02 ± 0.01[ab]	-	0.05 ±0.01[a]	0.001
4-Ethyl-2,6-dimethoxyphenol	0.13 ± 0.01[a]	0.10 ± 0.01[a]	0.10 ± 0.01[a]	0.11 ±0.01[a]	0.10 ±0.01[a]	0.394
2,6-Dimethoxy-4-propylphenol	0.05 ± 0.01[a]	0.03 ± 0.01[a]	0.03 ± 0.01[a]	0.02 ±0.01[a]	0.03 ±0.01[a]	0.100
4-Isopropylthiophenol	0.06 ± 0.01	-	-	-	-	-
2,5-Dimethyl-1,4-benzenediol	0.02 ± 0.01[c]	-	0.04 ± 0.01[a]	0.03 ±0.00[a]	-	< 0.0001
**Total**	21.17 ± 2.16[a]	10.03 ± 1.36[b]	7.38 ±0.13[b]	6.10 ±0.13[b]	5.15 ±0.07[b]	< 0.0001

**Ester (28)**						
Butanoic acid, methyl ester	-	0.43 ± 0.12	-	-	-	-
2,2-Dimethyl-butanoic acid, methyl ester	0.32 ± 0.01	-	-	-	-	-
Hexanoic acid, ethyl ester	0.22 ± 0.03	-	-	-	-	-
(Z)-9-Octadecenoic acid, hexyl ester	0.03 ± 0.001	-	-	-	-	-
Propanoic acid, 2-hydroxy-, ethyl ester	0.14 ± 0.02	-	-	-	-	-
Octanoic acid, methyl ester	0.04 ± 0.001	-	-	-	-	-
Octanoic acid, ethyl ester	0.18 ± 0.03[a]	-	0.07 ± 0.01[b]	0.04 ±0.001[b]	0.07 ±0.01[b]	< 0.0001
13-Tetradecynoic acid, methyl ester	-	-	0.04 ± 0.01[b]	0.06 ±0.01[a]	0.06 ±0.001[a]	< 0.0001
2-Nitro-ethanol, propionate (ester)	0.05 ± 0.001[a]	0.02 ± 0.001[b]	-	-	-	< 0.0001
Acetic acid, ethoxyhydroxy-, ethyl ester	6.98 ± 0.64	-	-	-	-	-
Pentanoic acid, 2-hydroxy-4-methyl-, ethyl ester	0.04 ± 0.001	-	-	-	-	-
Nonanoic acid, methyl ester	-	0.10 ± 0.01[a]	-	0.07 ±0.001[a]	-	< 0.0001
Formic acid, octyl ester	0.42 ± 0.07	-	-	-	-	-
Decanoic acid, methyl ester	0.39 ± 0.05[b]	0.96 ± 0.14[a]	-	-	0.09 ±0.01[b]	< 0.0001
Decanoic acid, ethyl ester	0.29 ± 0.02[a]	0.08 ± 0.001[b]	0.05 ± 0.01[b]	0.11 ±0.02[b]	0.07 ±0.001[b]	< 0.0001
Acetic acid, 2-(1-buten-3-yl)-2-nitro-, ethyl ester	0.12 ± 0.001	-	-	-	-	-
Formic Acid, 4-methoxyphenyl ester	-	0.07 ± 0.02	-	-	-	-
[1,1'-Bicyclopropyl]-2-octanoic acid, 2'-hexyl-, methyl ester	0.01 ± 0.001	-	-	-	-	-
9-Octadecenoic acid (Z)-, phenylmethyl ester	0.04 ± 0.001[b]	0.05 ± 0.001[a]	-	-	-	< 0.0001
2-Octyl-cyclopropanetetradecanoic acid, methyl ester	-	0.08 ± 0.02[a]	0.06 ± 0.02[ab]	-	0.01 ±0.001[b]	0.001
16-Octadecenoic acid, methyl ester	-	0.05 ± 0.001[a]	-	0.02 ±0.001[b]	-	< 0.0001
Methyl isovalerate	0.42 ± 0.03[a]	0.51 ± 0.30[a]	-	-	-	0.029
Butyric acid, 2-hydroxy-3-methyl-, methyl ester	0.01 ± 0.001	-	-	-	-	-
Methyl 12,13-tetradecadienoate	0.18 ± 0.03	-	-	-	-	-
E-2-Hexenyl benzoate	0.07 ± 0.01[a]	0.08 ± 0.03[a]	-	-	-	0.002

Hexadecanoic acid, methyl ester	-	-	-	0.02 ±0.001	-	-
Bicyclo[2.2.1]heptan-2-ol, 7,7-dimethyl-, acetate	0.02 ± 0.001	-	-	-	-	-
γ-Dodecalactone	0.10 ± 0.01	-	-	-	-	-
**Total**	10.18 ± 0.91[a]	2.48 ± 0.48[b]	0.23 ± 0.02[c]	0.36 ±0.01[c]	0.32 ±0.001[c]	< 0.0001
**Acids (13)**						
Trans-2-undecenoic acid	3.15 ± 0.36	-	-	-	-	-
Acetic acid	1.11 ± 0.13[a]	0.98 ± 0.31[a]	0.13 ± 0.02[b]	0.07 ±0.01[b]	-	< 0.0001
Muramic acid	0.08 ± 0.01	-	-	-	-	-
Lactic acid	0.11 ± 0.05	-	-	-	-	-
Butanoic acid	0.23 ± 0.02[a]	0.04 ± 0.01[b]	-	-	-	< 0.0001
3-Methyl-pentanoic acid	0.28 ± 0.03	-	-	-	-	-
3-Hydroxy-dodecanoic acid	0.02 ± 0.001	-	-	-	-	-
4-Oxo-4-(para-tolyl)-butyric acid	0.09 ± 0.02	-	-	-	-	-
Hexanoic acid	0.09 ± 0.03[a]	-	-	0.03 ±0.003[b]	-	< 0.0001
Bicyclo[2.2.1]heptane-1,2-dicarboxylic acid	0.19 ± 0.03[a]	0.02 ± 0.001[b]	-	-	-	< 0.0001
cis-7-Hexadecenoic acid	0.02 ± 0.004	-	-	-	-	-
Nonanoic acid	0.19 ± 0.007[ab]	0.38 ± 0.04[a]	0.25 ± 0.10[ab]	-	0.29 ±0.08[a]	0.005
n-Decanoic acid	0.27 ± 0.09[a]	-	-	0.18 ±0.02[ab]	0.13 ±0.02[ab]	0.001
**Total**	5.82 ± 0.56[a]	1.42 ± 0.29[b]	0.38 ± 0.09[b]	0.27 ±0.03[b]	0.42 ±0.09[b]	< 0.0001
**Ketones (16)**						
2-Methyl-2-Cyclopenten-1-one	0.34 ± 0.02	-	-	-	-	-
1-(2-Furanyl)-ethanone	0.13 ± 0.01[a]	0.07 ± 0.001[b]	-	-	-	< 0.0001
2-Ethyl-3-methylcyclopent-2-en-1-one	0.54 ± 0.6[a]	0.16 ± 0.04[b]	-	-	-	< 0.0001
2,3-Dimethyl-2-cyclopenten-1-one	0.43 ± 0.03[a]	0.11 ± 0.02[b]	0.07 ± 0.01[c]	-	-	< 0.0001
2-Butyl-3-methylcyclopent-2-en-1-one	0.04 ± 0.01	-	-	-	-	-
4,4a,5,6,7,8-Hexahydro-1-methoxy-2(3H)-naphthalenone	0.09 ± 0.001	-	-	-	-	-
2-(1-Methyl-2-nitroethyl)-cyclohexanone	0.04 ± 0.001[b]	0.25 ± 0.04[a]	-	0.10 ±0.01[b]	-	< 0.0001
Nona-3,5-dien-2-one	0.05 ± 0.001[a]	0.05 ± 0.001[a]	-	-	-	< 0.0001
2,4-Dimethyl-1,3-cyclopentanedione	0.15 ± 0.01[a]	0.09 ± 0.001[b]	0.02 ± 0.001[c]	0.01 ±0.001[c]	0.02 ±0.001[c]	< 0.0001
3-Methyl-1,2-cyclopentanedione	0.54 ± 0.04[a]	0.22 ± 0.05[b]	0.12 ± 0.08[b]	0.08 ±0.001[b]	0.09 ±0.01[b]	< 0.0001

2-(2-Methyl-propenyl)-cyclohexanone	0.05 ± 0.001[a]	0.02 ± 0.001[b]	-	-	-	< 0.0001
3-Ethyl-2-hydroxy-2-cyclopenten-1-one	0.14 ± 0.01[a]	0.07 ± 0.001[b]	0.06 ± 0.01[b]	0.04 ±0.001[c]	-	< 0.0001
2,3-Dihydro-3-methyl-1H-inden-1-one	0.01 ± 0.001	-	-	-	-	-
5-Heptyldihydro-2(3H)-furanone	0.05 ± 0.001	-	-	-	-	-
Acetoin	0.91 ± 0.08[a]	0.68 ± 0.15[a]	-	-	-	< 0.0001
5a-Methoxy-9a-methyl-3,4,5a,6,7,8,9a,10-octahydropyrano[4,3-b]chromene-1,9-dione	0.05 ± 0.001	-	-	-	-	-
**Total**	3.61 ± 0.26[a]	1.76 ± 0.30[b]	0.28 ± 0.03[c]	0.25 ±0.001[c]	0.11 ±0.01[c]	< 0.0001
**Alcohols (28)**						
Ethanol	0.22 ± 0.03[a]	0.18 ± 0.10[a]	0.09 ± 0.02[a]	0.06 ±0.01[a]	0.06 ±0.03[a]	0.122
(3-Methyl-oxiran-2-yl)-methanol	0.29 ± 0.03	-	-	-	-	-
4-Amino-1-butanol	0.06 ± 0.004	-	-	-	-	-
1-Pentanol	0.40 ± 0.05	-	-	-	-	-
3-Methyl-1-butanol	-	0.06 ± 0.003	-	-	-	-
2-Nitro-ethanol	0.12 ± 0.06[b]	0.19 ± 0.05[a]	-	-	-	0.002
(E)-2-Octen-1-ol	0.14 ± 0.02[a]	0.10 ± 0.02[a]	-	-	-	< 0.0001
Cyclohexanol	0.02 ± 0.003	-	-	-	-	-
1-Octen-3-ol	0.10 ± 0.02[a]	0.09 ± 0.004[a]	-	-	-	< 0.0001
1-Nonen-3-ol	-	-	0.04 ± 0.01[a]	0.02 ±0.003[b]	0.03 ±0.001[a]	0.001
2,3-Dimethyl-cyclohexanol	-	0.06 ± 0.02	-	-	-	-
2-Ethyl-1-hexanol	0.08 ± 0.01[a]	0.05 ± 0.004[b]	-	-	-	< 0.0001
2,3-Butanediol	0.12 ± 0.04[a]	0.14 ± 0.01[a]	-	-	-	< 0.0001
3-Methyl-2-butanol	0.14 ± 0.02	-	-	-	-	-
Linalool	-	0.06 ± 0.01	-	-	-	-
5-Amino-pentanol	0.07 ± 0.003	-	-	-	-	-
1-Nonanol	0.28 ± 0.04[a]	0.12 ± 0.01[b]	-	-	-	< 0.0001
2-Furanmethanol	0.47 ± 0.04[a]	0.09 ± 0.02[b]	0.01 ±0.001[b]	-	-	< 0.0001
1-Decanol	-	0.32 ± 0.07[a]	0.20 ± 0.02[ab]	0.14 ±0.01[bc]	0.14 ±0.01[bc]	< 0.0001
(E)-2-Decen-1-ol	-	-	0.03 ±0.01[b]	0.05 ±0.002[a]	-	< 0.0001
5-Methyl-2-furanmethanol	0.16 ± 0.02[a]	0.04 ± 0.003[b]	-	-	-	< 0.0001
3-Quinuclidinol	0.04 ± 0.003[a]	0.03 ± 0.003[b]	0.01 ± 0.001[c]	-	-	< 0.0001
1-Benzofuran-5-ol	0.04 ± 0.004[a]	-	0.03 ± 0.004[b]	0.03 ±0.002[ab]	-	< 0.0001
2-Octanol	0.02 ± 0.001	-	-	-	-	-

1-Ethynyl-1-cycloheptanol	0.24 ± 0.02	-	-	-	-	-
2-Methyl-4-heptanol	0.07 ± 0.01	-	-	-	-	-
4-Ethyl-1,3-benzenediol	-	0.04 ± 0.01[a]	0.02 ± 0.01[b]	0.01 ±0.002[bc]	0.01 ±0.001[bc]	< 0.0001
p-Cymene-2,5-diol	0.03 ± 0.003	-	-	-	-	-
**Total**	3.08 ± 0.28[a]	1.55 ± 0.19[b]	0.43 ± 0.05[c]	0.31 ±0.03[c]	0.24 ±0.03[c]	< 0.0001
**Aromatic compounds (7)**						
p-Xylene	0.67 ± 0.06[a]	0.23 ± 0.06[b]	0.15 ± 0.01[b]	0.14 ±0.01[b]	0.18 ±0.001[b]	< 0.0001
1,2-Dimethoxy-benzene	0.24 ± 0.02[a]	0.09 ± 0.01[b]	-	-	-	< 0.0001
3,4-Dimethoxytoluene	0.28 ± 0.04[a]	0.09 ± 0.02[b]	0.06 ± 0.01[b]	0.05 ±0.01[b]	-	< 0.0001
1,2,3-Trimethoxybenzene	0.08 ± 0.001[a]	0.02 ± 0.003[b]	0.02 ± 0.001[b]	-	-	< 0.0001
2,4-Dimethoxytoluene	0.03 ± 0.004	-	-	-	-	-
1,4-Dimethoxy-2,3-dimethylbenzene	0.01 ± 0.001	-	-	-	-	-
3,5-Dimethoxy-4-hydroxytoluene	0.71 ± 0.07[a]	0.36 ± 0.05[b]	0.48 ± 0.01[b]	0.44 ±0.02[b]	0.38 ±0.04[b]	< 0.0001
**Total**	2.03 ± 0.19[a]	0.81 ± 0.10[b]	0.72 ± 0.03[b]	0.64 ±0.02[b]	0.56 ±0.04[b]	< 0.0001
**Furan (3)**						
2,2,4,4-Tetramethyl-tetrahydro-furan	0.04 ± 0.01	-	-	-	-	-
Furfural	0.34 ± 0.04[a]	-	0.03 ± 0.001[b]	0.06 ±0.001[b]	0.01 ±0.001[b]	< 0.0001
2-Acetyl-5-methylfuran	0.14 ± 0.02[a]	-	0.01 ± 0.001[b]	-	-	< 0.0001
**Total**	0.54 ± 0.05[a]	-	0.04 ± 0.001[b]	0.06 ±0.001[b]	0.01 ±0.001[b]	< 0.0001
**Aldehydes (7)**						
Hexanal	0.17 ± 0.03[a]	-	0.03 ± 0.003[b]	0.06 ±0.003[b]	0.08 ±0.01[b]	< 0.0001
Octanal	0.23 ± 0.03	-	-	-	-	-
Nonanal	-	-	0.53 ± 0.12[b]	0.82 ±0.06[a]	0.75 ±0.05[ab]	< 0.0001
2,5-Dihydroxybenzaldehyde, 2TMS derivative	-	0.03 ± 0.001	-	-	-	-
Hexadecanal	0.04 ± 0.004[a]	-	0.03 ± 0.002[a]	-	0.04 ±0.002[a]	< 0.0001
Benzeneacetaldehyde	0.15 ± 0.03	-	-	-	-	-
2-Formyl-3-methyl-α-methylene-cyclopentaneacetaldehyde	0.04 ± 0.01	-	-	-	-	-

**Total**	0.64 ± 0.08[a]	0.03 ± 0.01[c]	0.59 ± 0.12[b]	0.88 ±0.06[a]	0.87 ±0.06[a]	< 0.0001
**Hydrocarbons (5)**						
(R)-1-Methyl-5-(1-methylethenyl)-cyclohexene	-	2.90 ± 0.88[a]	0.36 ± 0.12[b]	-	-	< 0.0001
1-Tetradecene	0.22 ± 0.02[a]	0.12± 0.02[b]	0.08 ± 0.01[b]	0.07 ±0.01[b]	0.07 ±0.01[b]	0.001
Azulene	0.05 ± 0.001	-	-	-	-	-
1-Methyl-3-(1-methylethyl)-cyclohexene	-	0.12 ± 0.03	-	-	-	-
1,5,5-Trimethyl-6-methylene-cyclohexene	0.07 ± 0.001	-	-	-	-	-
**Total**	0.35 ± 0.03[b]	3.16± 0.91[a]	0.45 ± 0.14[b]	0.07 ±0.01[b]	0.07 ±0.01[b]	< 0.0001
**Other (19)**						
Formamide, N-methylthio	-	0.04 ± 0.01[a]	0.02 ± 0.00[b]	-	-	< 0.0001
N-[S-Benzylcysteinyl]-S-[2-tetrahydropyranyl]cysteine amide	0.07 ± 0.00[a]	0.03 ± 0.01[b]	-	-	-	< 0.0001
3,N-Dihydroxy-butanamide	-	0.14 ± 0.03	-	-	-	-
Hydroperoxide, hexyl	0.07 ± 0.01	-	-	-	-	-
(Z)-1-Ethoxy-2-heptene	0.08 ± 0.01	-	-	-	-	-
Paromomycin	0.02 ± 0.01[b]	0.08 ± 0.01[a]	0.07 ± 0.01[a]	-	-	< 0.0001
Trichloromethane	0.31 ± 0.04[a]	0.07 ± 0.01[b]	0.10 ± 0.01[b]	0.13 ±0.02[b]	0.09 ±0.02[b]	< 0.0001
(1R,3E,7E,11R)-1,5,5,8-Tetramethyl-12-oxabicyclo[9.1.0]dodeca-3,7-diene	0.03 ± 0.02	-	-	-	-	-
12-Crown-4	0.03 ± 0.01	-	-	-	-	-
Tetrahydro-2-(2-propynyloxy)-2H-pyran	0.05 ± 0.01	-	-	-	-	-
3-Trifluoroacetoxypentadecane	-	0.05 ± 0.01	-	-	-	-
9-Hydroxy-9-borabicyclo[3.3.1]nonane	0.10 ± 0.02[b]	0.14 ± 0.05[a]	-	-	-	0.003
Methoxy-phenyl-oxime	0.16 ± 0.03[a]	0.24 ± 0.13[a]	-	-	-	0.027
1-Acetyl-1,2-epoxy-cyclopentane	0.10 ± 0.01[a]	0.07 ± 0.01[b]	0.04 ± 0.001[c]	-	-	< 0.0001
4-Cyano-5-ethyl-2-methoxy-6-phenylpyridine-3-carboxamide	0.01 ± 0.01	-	-	-	-	-
2-(2-Isopropenyl-5-methyl-cyclopentyl)-acetamide	-	0.02 ± 0.00	-	-	-	-

cis-1-ethylideneoctahydro-7a-methyl-1H-Indene	0.01 ± 0.01	-	-	-	-	-
6-Methyl-1H-indole	0.01 ± 0.001^{a}	-	-	0.02 ± 0.01^{a}	0.02 ± 0.00^{a}	< 0.0001
Octaethylene glycol monododecyl ether	0.02 ± 0.00^{b}	-	0.04 ± 0.01^{a}	0.03 ± 0.001^{b}	0.02 ± 0.001^{b}	0.038
**Total**	1.17 ± 0.07^{a}	0.92 ± 0.08^{b}	0.29 ± 0.03^{c}	0.19 ± 0.03^{c}	0.13 ± 0.05^{c}	< 0.0001

Data are expressed as means ± SE (n = 4).

$^{a, b}$Means with different superscripts within the rows are significantly different ($p < 0.05$).
